# Evidence from bioinformatics, expression and inhibition studies of phosphoinositide-3 kinase signalling in *Giardia intestinalis*

**DOI:** 10.1186/1471-2180-6-45

**Published:** 2006-05-18

**Authors:** Siân SE Cox, Mark van der Giezen, Sarah J Tarr, Mark R Crompton, Jorge Tovar

**Affiliations:** 1 School of Biological Sciences, Royal Holloway University of London, Egham, Surrey, UK; 2 School of Biological and Chemical Sciences, Queen Mary, University of London, Mile End Road, London, UK

## Abstract

**Background:**

*Giardia intestinalis *is a parasitic protozoan and major cause of diarrhoeal disease. Disease transmission is dependent on the ability of the parasite to differentiate back and forth between an intestine-colonising trophozoite and an environmentally-resistant infective cyst. Our current understanding of the intracellular signalling mechanisms that regulate parasite replication and differentiation is limited, yet such information could suggest new methods of disease control. Phosphoinositide-3 kinase (PI3K) signalling pathways have a central involvement in many vital eukaryotic processes, such as regulation of cell growth, intracellular membrane trafficking and cell motility. Here we present evidence for the existence of functional PI3K intracellular signalling pathways in *G. intestinalis*.

**Results:**

We have identified and characterised two genes, *Gipi3k1 *and *Gipi3k2*, which encode putative PI3Ks. Both genes are expressed in trophozoites and encysting cells, suggesting a possible role of GiPI3K1 and GiPI3K2 in regulating giardial growth and differentiation. Extensive nucleotide and amino acid sequence characterisation predicts that both encoded PI3Ks are functional as indicated by the presence of highly conserved structural domains and essential catalytic residues. The inhibitory effect of the PI3K inhibitor LY294002 on trophozoite proliferation also supports their functionality. Phylogenetic analysis supports the identity of GiPI3K1 as a Class I isoform and GiPI3K2 as a Class III isoform. In addition, giardial genes encoding putative homologues of phosphoinositide-metabolising enzymes such as PTEN, MTM, PIPkin and PI 5-phosphatase as well as downstream effectors with phosphoinositide-binding domains have been identified, placing GiPI3K1 and GiPI3K2 in a broader signalling context. Compared with twenty-six PI3Ks from other organisms, GiPI3K1 and GiPI3K2 are unique in that they contain large insertions within their highly conserved kinase domains. The function of these insertions is unknown; however we show here that they are not intron-derived and would probably not hinder substrate binding. These insertions may represent a plausible drug target.

**Conclusion:**

*G. intestinalis *encodes and expresses two putative PI3Ks, at least one of which appears to be required during normal parasite proliferation. The identification of Class I and Class III but not Class II isoforms suggests that both extracellularly-initiated signalling (Class I-regulated) and intracellular vesicle trafficking (Class III-regulated) might be controlled by PI3Ks in *G. intestinalis*. The presence of genes encoding putative homologues of phosphoinositide-metabolising enzymes and downstream effectors in the *G. intestinalis *genome further suggests that the overall architecture of PI3K signalling may be comparable with pathways present in other better-studied organisms.

## Background

The parasitic protozoan *Giardia intestinalis *(syn. *lamblia, duodenalis*) is a common cause of water-borne diarrhoeal disease, particularly in the developing world. *G. intestinalis*'s infective ingenuity stems from its ability to switch between two morphologically distinct states: an externally protected, inert cyst and a flagellated colonizing trophozoite that can attach to host cells in the small intestine. This attachment leads to the unpleasant symptoms associated with giardiasis [1]. Differentiation of *G. intestinalis *has been studied extensively in terms of analysing the morphological events associated with encystation (trophozoite-to-cyst) and excystation (cyst-to-trophozoite) [1], yet little is known about the molecular circuitry that regulates differentiation. Although limited in their number, a few studies have given some crucial insights into the intracellular signalling pathways that may allow the organism to respond to environmental cues. For instance, protein kinase A and cyclic-AMP, in addition to calcium and calmodulin have been shown to play an important role in trophozoite motility and initiation of excystation [2-4] while catalytically active homologs of the intracellular signalling mitogen-activated protein kinase family in *G. intestinalis *appear to be important for growth and encystation [5]. The potential diversity of intracellular signalling mechanisms in *G. intestinalis *is illustrated by the recent characterisation of a giardial protein kinase B-like molecule [6] and by the demonstration that cholesterol starvation *in vitro *induces *Giardia *encystation [7]. The latter suggests a role for lipids and lipid signalling in parasite differentiation.

Phosphatidylinositol (PtdIns) is a basic building block for intracellular inositol lipids in eukaryotic cells and is present in trace amounts in *G. intestinalis *trophozoites and encysting cells cultured *in vitro *[8]. It has been shown that *G. intestinalis *uses inositol headgroup exchange enzymes to convert free inositol to PtdIns [9]. In *Dictyostelium discoideum*, yeast and mammalian cells, PtdIns can be reversibly phosphorylated at the D-3 position of its inositol ring by phosphoinositide 3-kinases (PI3Ks), and at other positions by distinct kinases, to form phosphorylated PtdIns; PtdIns and its phosphorylated derivatives are collectively referred to as phosphoinositides (PIs) [10]. Although PIs constitute a minor fraction of total cellular lipids in eukaryotic cells, they are biologically important as second messengers and their levels must be accurately regulated [11].

Although PtdIns has been detected in *Giardia *[8] it is unclear whether this parasite utilises PI3K-mediated intracellular signalling mechanisms to regulate its growth and differentiation. PI3K signalling is associated with many eukaryotic events such as regulation of cell growth, intracellular membrane trafficking and cell motility [12], each of which is important in the lifecycle of *G. intestinalis*. PI3K signalling has been extensively characterised in mammals, yeast and most notably in the slime mold *Dictyostelium discoideum*, where several PI3Ks are known to be important for normal cell growth, multicellular development, aggregation and chemotaxis [13,14].

PI3Ks become activated in response to numerous agonists and the resulting PIs initiate the recruitment of signalling proteins to membranes – specifically by way of PI-binding domains present on the proteins. In this way, membrane signals can be spatially restricted and highly controlled. A well-characterised example of a PI-binding signalling protein is protein kinase B (PKB), which, when membrane-recruited by PI, is only then able to phosphorylate specific substrates in order to regulate apoptosis, protein synthesis, cell growth and cell cycling [12]. To date, eight mammalian PI3Ks have been identified and they comprise three classes of isozymes on the basis of sequence similarity and substrate preference *in vivo *[11]. Class I PI3Ks phosphorylate PtdIns, PtdIns(4)P and PtdIns(4,5)P_2_, Class II PI3Ks phosphorylate PtdIns and PtdIns(4)P but not PtdIns(4,5)P_2 _and Class III PI3Ks (sometimes referred to as Vps34, after the isoform detected in yeast [15]) exclusively phosphorylate PtdIns. All PI3Ks characterised to date have a multi-domain structure, comprising a highly conserved two-lobed catalytic kinase domain at their C-terminus, a PIK domain which acts as a scaffold around which the enzyme is folded and a C2 or C2-like domain that in other proteins binds phospholipids and so may be involved in substrate targeting. The PI3Ks also have domains that mediate enzyme activation and promote enzyme interaction with the plasma membrane, namely the Ras Binding Domains (RBDs) common to Class I and II isoforms, and adaptor protein binding domains [11].

Using mammalian and yeast PI3K protein sequences as queries, we searched the *G. intestinalis *genome [16] to identify putative molecular homologues. Here, we report on the characterisation of two giardial genes, which we term *Gipi3k1 *and *Gipi3k2*, encoding putative PI3Ks. The presence of well conserved structural PI3K domains in these sequences and the detection of specific transcripts for both genes in trophozoites and encysting cells suggest that GiPI3K1 and GiPI3K2 are functional enzymes. Functionality of at least one of the putative PI3Ks is experimentally demonstrated by the specific and significant inhibitory effect of a PI3K inhibitor on trophozoite proliferation. We further substantiate the existence of PI3K signalling in *G. intestinalis *by identifying additional homologs which may be important for the metabolism and downstream effects of the giardial PI3K enzymatic products.

## Results

### Identification and sequence characterisation of two putative *G. intestinalis *PI3K genes

Using catalytic domain sequences of Class I, II and III PI3Ks as query sequences, we carried out sequence similarity searches to identify *Giardia *genes encoding putative PI3Ks. Our searches returned several open reading frames, two of which demonstrated similarity to PI3K genes (Table 1) and were therefore called *Gipi3k1 *and *Gipi3k2*. The predicted open reading frames of *Gipi3k1* and *Gipi3k2* are 6,468 bp and 4,917 bp in length respectively. *Gipi3k1* is predicted to encode a 2,155 amino acid protein [GenPept: EAA41385] with a molecular mass of 242 kDa, whilst *Gipi3k2* is predicted to encode a 1,638 amino acid protein [GenPept: EAA40923] with a molecular mass of 183 kDa. We independently confirmed the entire *Gipi3k1 *sequence given by the *G. intestinalis *genome project, (see Methods section) and no differences in sequence were observed.

**Figure 1:**
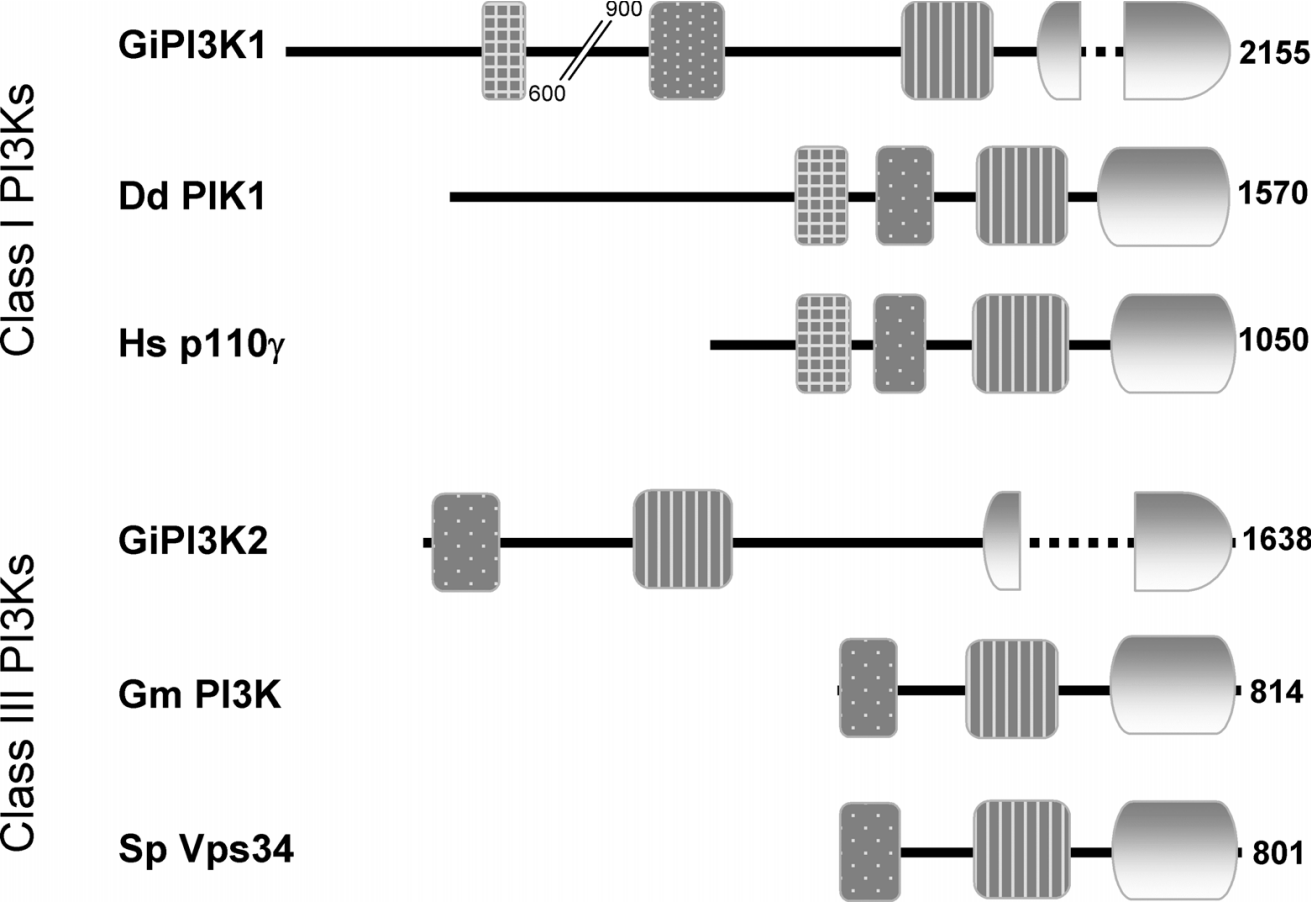
**Domain structure of GiPI3K1 and GiPI3K2 compared with other PI3Ks**. Top: GiPI3K1 is compared to Dd PIK1, *Dictyostelium discoideum *Class I PI3K [GenPept: AAA85721] and to Hs p110γ, *Homo sapiens *Class I PI3K [GenPept: NP_002640]. The GiPI3K1 sequence is interrupted for the sake of clarity. Bottom: GiPI3K2 is compared to Gm PI3K, *Glycine max *Class III PI3K [GenPept: AAA64468] and to Sp Vps34, *Schizosaccharomyces pombe *Class III PI3K [GenPept: AAC49133]. Numbers indicate amino acids. Domains are shaded as follows: checked, Ras binding; dotted, C2; striped, PIK; gradient, kinase. Apparent insertions in the giardial kinase domains are indicated by a dotted line.

PI3K – characteristic domains were predicted to be present and in the typical conformation for both GiPI3K1 and GiPI3K2 (Figure 1). Ubiquitin/RBD-like, C2, PIK and kinase domains were predicted for GiPI3K1 with significant expectant (E) values (i.e. the E-values were below the default threshold set for significance). C2, PIK and kinase domains were predicted for GiPI3K2, again with significant E-values. Interestingly, both putative kinase domains appeared to be interrupted by long insertions as compared with other well-studied PI3Ks (see Figure 1, dotted lines). We carried out sequence-similarity searches with the predicted Ubiquitin/RBD-like domain of GiPI3K1 which returned matches against the Ras Binding Domains of several mammalian Class I PI3Ks (see Table 1 for similarity scores). This finding is interesting, since *G. intestinalis *apparently contains no Ras protein-encoding sequences [17]. A recent (May 2006, unpublished) *G. intestinalis *genome survey by us supports this claim: we could only identify putative Rab, Ran and Rac GTPases encoded in the *G. intestinalis *genome [16], but no Ras-encoding sequences. One could speculate that the putative RBD of GiPI3K1 may be recruited to membranes by another Ras-like small GTPase such as Rab.

**Figure 2:**
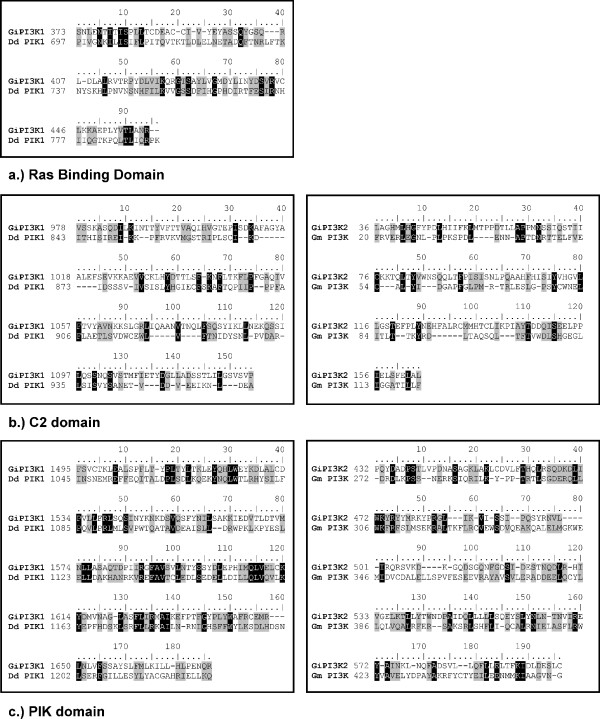
**PI3K domain pairwise alignments for GiPI3K1 and GiPI3K2**. Pairwise sequence alignment of GiPI3K1 and GiPI3K2 indicates conservation in predicted Ras Binding (a), C2 (b) and PIK (c) domains. Identical aligned residues are indicated in black shading, while conserved residues are indicated by grey shading according to the PAM250 matrix [45]. Identity/similarity percentages for the GiPI3K1 alignments are 15%/45%, 12%/31% and 20%/51% for the GiPI3K1 RBD, C2 and PIK domains respectively, for the GiPI3K2 alignments these values are 13%/31% and 16%/47% for the GiPI3K2 C2 and PIK domains respectively.

Full-length and domain-only sequence similarity analysis of GiPI3K1 and GiPI3K2 demonstrated clear similarity with PI3Ks from other organisms, in particular GiPI3K1 is predicted to be similar to Class I PI3Ks and GiPI3K2 to Class III PI3Ks (Table 1, see also next section). Due to low conservation of sequence similarity amongst C2 domains from various PI3Ks, similar analysis carried out for the predicted C2 domains of GiPI3K1 and GiPI3K2 did not return significant matches to the C2 domains of other PI3Ks. However, C2 pairwise sequence alignments (Figure 2) demonstrated reasonable C2 domain similarity.

To further substantiate the identity of the giardial PI3K domains, pairwise alignments of the RBD of GiPI3K1 and the C2 and PIK domains of GiPI3K1 and GiPI3K2 were carried out with PI3Ks which shared the greatest similarity with the giardial PI3Ks (see Table 1). Figure 2 shows how the domains of GiPI3K1 and GiPI3K2 align well with domains of PI3Ks from *D. discoideum *and *Glycine max *respectively.

**Figure 3:**
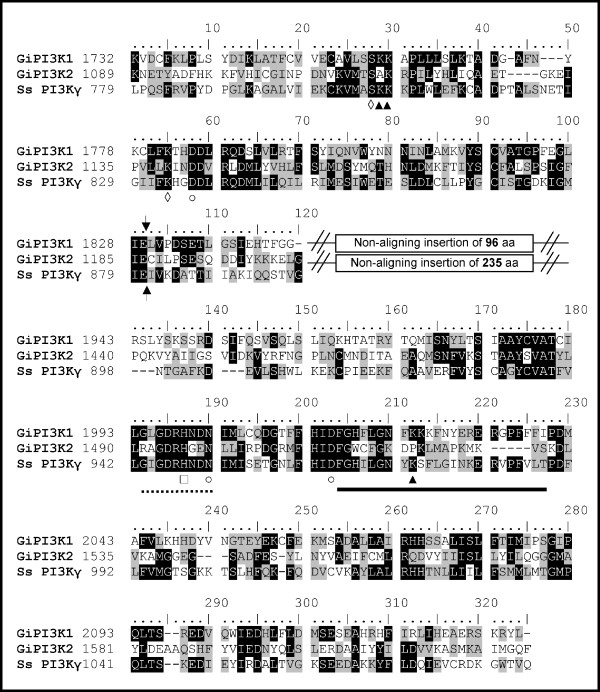
**Detailed sequence analysis of the kinase domains of GiPI3K1 and GiPI3K2**. Multiple sequence alignment of the kinase domains of the giardial PI3Ks with a structurally characterised Class I PI3K-kinase domain from *Sus scrofa*, Ss PI3Kγ, [GenPept: NP_999104] demonstrates conservation of functional residues. Sequences were aligned using ClustalW [37]. Arrows indicate the boundary between the N- and C-terminal lobes of the kinase domain for the *S. scrofa *sequence. Insertions in the kinase domains of GiPI3K1 and GiPI3K2 are shown in boxes (not to scale). Solid underlined residues indicate activation loop residues in the *S. scrofa *sequence essential for substrate specificity. Dashed underlined residues indicate catalytic loop residues in the *S. scrofa *sequence. Key: ◇, ATP binding residues; ▲, PI-binding residues; ○, metal binding residues; □, possible substrate-activating base/nucleophile to attack γ-phosphate of ATP.

A similar, detailed multiple alignment analysis was carried out for the kinase domains of GiPI3K1 and GiPI3K2 against the structurally characterised kinase domain of PI3Kγ (a Class IB PI3K) from the domestic pig, *Sus scrofa *[18] (Figure 3). All residues required for ATP binding and kinase activity in the *S. scrofa *PI3Kγ are conserved in both GiPI3K1 and GiPI3K2. However, GiPI3K2 lacks one of the lysines thought to interact with the I-phosphate of PI substrate (Lys-807 in the *S. scrofa *sequence) and a lysine thought to act as a ligand for the 5-phosphate of PI(4,5)P_2 _(Lys-973 in the *S. scrofa *sequence) [18]. In addition, GiPI3K2's activation loop appears to be shorter. All of these features are common to other functional Class III PI3K isoforms such as *D. discoideum *DdPIK5 [13], which are thought to be specific for PtdIns only since their short activation loops cannot accommodate the 4-phosphate of PtdIns(4)P substrate [18]. GiPI3K1's activation loop is similar in length to that of the *S. scrofa *PI3Kγ. Taken together, this analysis strongly suggests that GiPI3K1 and GiPI3K2 are functional PI3Ks belonging to the Class I and Class III isoforms respectively.

**Figure 4:**
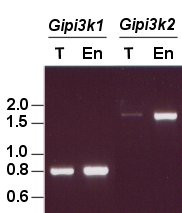
**RT-PCR analysis of *Gipi3k1 *and *Gipi3k2***. Purified RNA extracted from *G. intestinalis *trophozoites (T) and 36 hr encysting cells (En) was converted to cDNA via a reverse transcriptase (RT) reaction and subjected to PCR to amplify regions encoding the apparent insertions in GiPI3K1 and GiPI3K2. The resulting PCR products were separated on an agarose gel. Minus RT controls were also carried out to ensure product originated from RNA only (not shown). Molecular size markers are in kilobases.

Both the kinase domains of GiPI3K1 and GiPI3K2 have long insertions (96 and 235 amino acids in length respectively) that do not align with sequences in the kinase domain of *S. scrofa *PI3Kγ, and neither do they align with each other. The positions of these insertions are intriguing since they are close to the highly conserved catalytic loop residues, and appear to sit between the N- and C-terminal lobes of the predicted kinase domains (Figure 3). Secondary structure predictions of the insertions indicate that they may fold into additional structures associated with the respective kinase domains. *In silico *three-dimensional structural sequence alignments using the structurally characterised *S. scrofa *PI3Kγ as a template suggest that the insertions are probably surface-exposed with respect to the two lobes of the kinase domains, aligning to a coil between two alpha helices (data not shown). Thus, these putative domains could be accommodated without preventing the correct folding of the kinase domains and may not hinder substrate access. Sequence similarity searches of the insertions did not yield any evidence in terms of predicting their biological role. Multiple alignments with twenty-six Class I and Class III PI3Ks from different organisms indicated that the apparent insertions are a unique feature of the giardial sequences. These observations prompted speculation as to the nature of the insertions. Because trophozoites undergo substantial morphological changes and alterations in gene expression [19] the insertions could be subjected to differential splicing – perhaps as a way of controlling enzyme activity [20]. However, RT-PCR experiments using 36 hr encysting cell RNA provided no evidence for the splicing of the insertion-encoding sequences of *Gipi3k1 *or *Gipi3k2 *RNA during parasite differentiation (Figure 4).

### Phylogenetic analysis of GiPI3K1 and GiPI3K2

**Figure 5:**
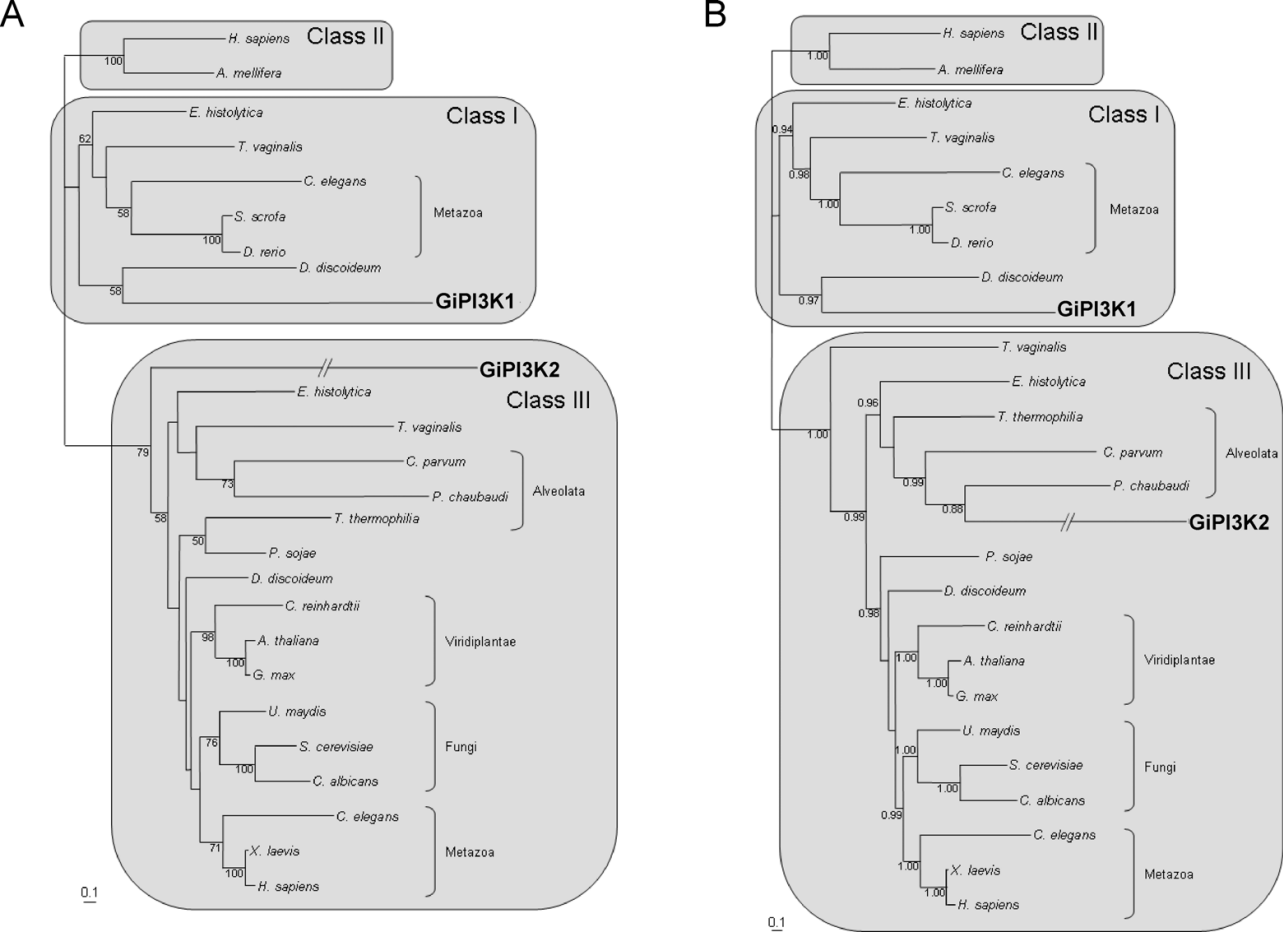
**Phylogenetic analysis of *G. intestinalis *PI3K homologs**. Phylogenetic analyses of the different classes of PI3Ks including GiPI3K1 and GiPI3K2 (shown in the Class I and Class III clusters respectively). Shown are unrooted trees of representative sequences of Class I, Class II and Class III PI3Ks. A, Maximum likelihood analysis using PHYML [40]. Bootstrap values based on 100 replicates indicated at the nodes, only values above 50% are shown. B, Bayesian analysis of the same dataset using MrBayes v3.1 [39], posterior probabilities, calculated by MrBayes are indicated at the nodes, only values above 0.75 are shown. The branches leading to GiPI3K2 have been shortened by one third.

Phylogenetic analyses of the two putative giardial PI3Ks clearly show their distinctive nature (Figure 5). GiPI3K1 clusters with the well characterised *D. discoideum *PIK1. Together, they are part of a clade exclusively containing PI3K1 homologs further strengthening our prediction that GiPI3K1 is a Class I isoform. The GiPI3K2 sequence belongs to a well-supported clade containing only Class III homologs, in agreement with our prediction based on domain composition (see Figure 1 and previous section). Although crown taxa fall in well-supported clades for the Class III homologs, support for the placement of the other sequences is lacking. The *G. intestinalis *Class III homolog is either basal to all other Class III homologs (Fig. 5A) or within the alveolates (Fig. 5B). The lengths of the branches leading to the *G. intestinalis *homologs are the longest in the analyses indicative of an accelerated evolution of these sequences. Phylogenetic reconstruction artefacts such as Long Branch Attraction [21] are therefore expected to affect these analyses. This would most likely only affect the placement within the isoform clade but not across isoforms. Therefore, our analyses cannot say much about the evolution of the *G. intestinalis *PI3Ks but can be used to identify which isoform each sequence belongs to.

### Characterisation of *Gipi3k1 *and *Gipi3k2 *gene products

**Figure 6:**
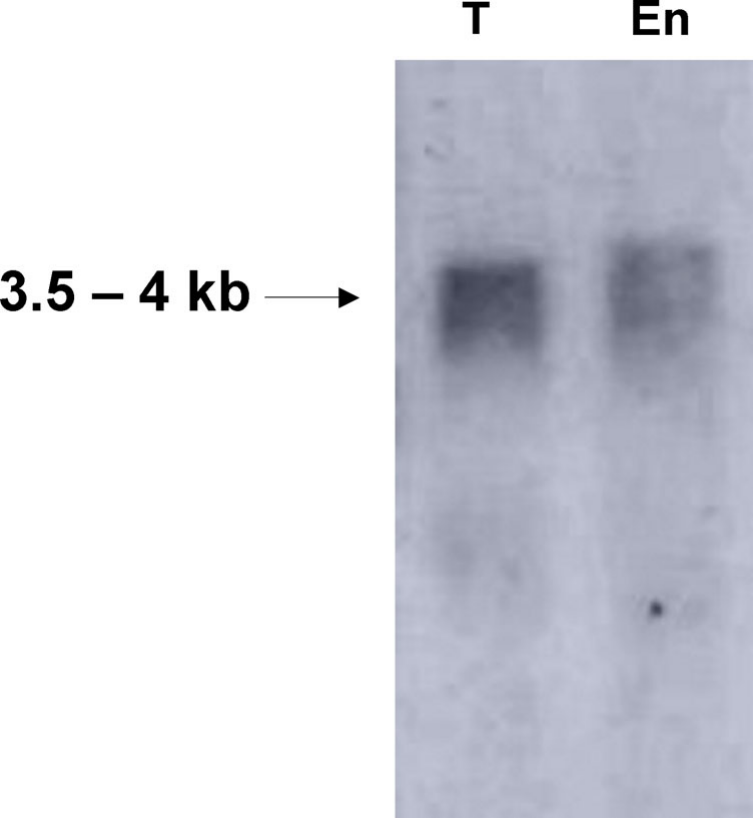
**Northern blot of *Gipi3k1 *RNA in trophozoites and encysting cells**. Northern blotting of trophozoite (T) and encysting cell (En) total RNA, as described in the Methods section, was carried out using a probe specific to *Gipi3k1 *RNA sequence to identify transcripts of approximately 3.5 – 4 kilobases.

**Figure 7:**
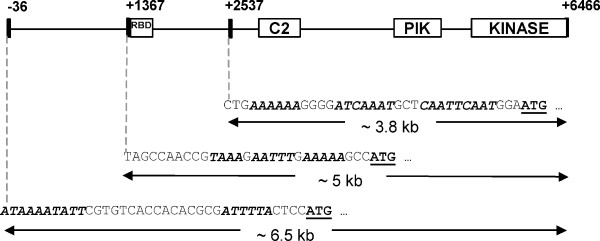
**Analysis of transcriptional initiation sites of the *Gipi3k1 *gene**. 5' RACE analysis, performed as described in the Methods section, suggests the presence of multiple transcription initiation sites for the *Gipi3k1 *gene. Sequence motifs common in *G. intestinalis *promoters are in italics and bold. Putative start codons are in bold and underlined. Predicted sizes of three *Gipi3k1 *mRNA transcripts are shown.

RT-PCR analysis (Figure 4) demonstrated that *Gipi3k1 *and *Gipi3k2 *are both expressed in trophozoites and 36 hr encysting cells, suggesting they play an important role during growth and possibly during encystation. The predicted open reading frames of *Gipi3k1 *and *Gipi3k2 *encode longer amino acid sequences as compared with other well-studied Class I and III PI3Ks (Figure 1). To test the coding capacity of these genes, we probed northern blots of RNA derived from trophozoites and 36 hr encysting cells with sequences specific to the highly conserved kinase domain-encoding region of *Gipi3k1 *and *Gipi3k2*. We were unable to detect *Gipi3k2 *mRNA despite using control probes against well-characterised giardial transcripts to confirm RNA integrity (data not shown). This may indicate that the *Gipi3k2 *message is relatively unabundant, detectable only by sensitive techniques such as RT-PCR. The apparent *Gipi3k1 *mRNA transcript detected by northern blotting (Figure 6) was smaller than expected based on the predicted size of the *Gipi3k1 *open reading frame: 3.5 – 4 kb as opposed to ~6.5 kb. Subsequent 5'RACE experiments (Figure 7) indicated that three transcriptional initiation sites of the *Gipi3k1 *gene may exist, capable of producing transcripts of ~6.5 kb, ~5kb, and ~3.8 kb in length, assuming they shared a common 3' polyadenylation site. A typical giardial polyadenylation site conforming to the consensus *AGTPurAAPyr *[22] for the *Gipi3k1 *gene was found (AGTATAAT), however putative polyadenylation sites for *Gipi3k2 *could not be identified. The shortest *Gipi3k1 *5'RACE product could represent the *Gipi3k1 *transcript detected by northern blotting. This suggests that the major transcript of the *Gipi3k1 *gene would encode a product more akin to the molecular weights observed for other well-studied PI3Ks [11], although it would lack the potential RBD. This may imply the existence of protein products which not only differ in size but also in the way in which they are regulated. The RBD of a Class I PI3K has been shown to stimulate the PI3K's kinase activity in addition to stabilising its association with the plasma membrane [23]. Thus *Gipi3k1 *protein products with an RBD may display differences in activity and/or localisation compared to those without an RBD.

Possible giardial promoter-like elements found upstream from each of the three alternative *Gipi3k1 *transcripts are also indicated in Figure 7. Promoter elements in *G. intestinalis *tend to have a relatively loose consensus, however it is generally thought that an AT-rich initiator of 8–10 bp exists near the transcription/translation start site in addition to a longer AT-rich element further upstream from the transcriptional start site which may contain CAAT boxes [24]. It is interesting that the apparent promoters of all three *Gipi3k1 *transcripts have some or all of these features. In addition, the shortest transcript is the only one to have upstream CAAT boxes. Possibly the 'loose' molecular machinery controlling giardial transcription [25] could be responsible for the presence of three *Gipi3k1 *transcripts, whereby transcription is initiated at several AT-rich regions. The use of specific antibodies against GiPI3K1 will be required to clarify whether the transcripts encode multiple proteins with distinct functions.

### Effect of PI3K inhibition on trophozoite proliferation

**Figure 8:**
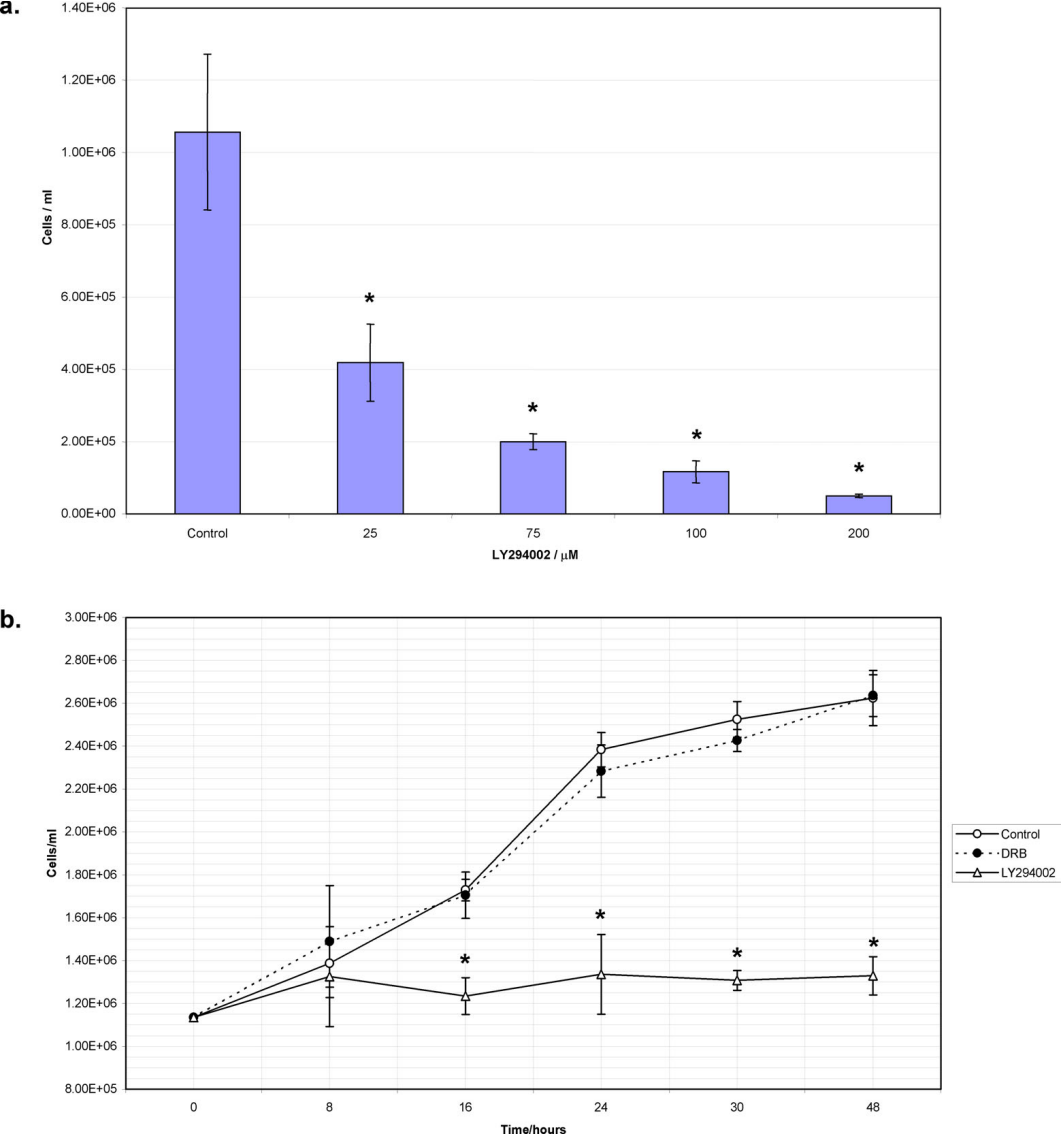
**Inhibition of trophozoite proliferation by a PI3K inhibitor**. a) Trophozoites treated with increasing concentrations of LY294002 were counted after 48 hours of treatment. b) To test the specificity of inhibition, the effect of LY294002 (50 μM) and DRB (100 μM final concentration) on exponentially growing trophozoites was monitored by cell counting over a 48 hour period. Error bars indicate the standard deviation of 3 counts. Asterisks denote significance at p < 0.001 for normalised data.

To determine the functional role of putative PI3Ks in *G. intestinalis *growth, we applied a commonly used PI3K inhibitor, LY294002, on *G. intestinalis *trophozoites. We tested a range of concentrations around those shown to selectively inhibit mammalian PI3Ks [26]. Figure 8a shows a dose-response of exponentially growing cells to the inhibitor, with concentrations of LY294002 as low as 25 μM causing a significant inhibitory effect on cell number as compared with the untreated control. Approximately 50% inhibition of cell proliferation occurred at concentrations between 25 and 75 μM. This effect is likely to be PI3K-mediated, since LY294002 concentrations within the 50 – 100 μM range have been employed for selective PI3K inhibition in mammalian cells. To understand the time course over which LY294002 exhibited its effects, we counted 50 μM LY294002 – treated cells at regular intervals over a 48 hour period. Figure 8b demonstrates that LY294002 begins to significantly effect cell number 8 hours into treatment. For the duration of the time-course, cell number remains approximately constant, whilst the untreated control continues to grow exponentially. This suggests that LY294002 may affect cell proliferation by inducing cell cycle arrest. In addition, trophozoites treated with LY294002 do not undergo any dramatic changes in their morphology or motility, thus further demonstrating the selective effect of LY294002 on *Giardia*'s cell cycle.

To ensure that the effects of LY294002 were due to inhibition of one or more of the putative PI3Ks and not another target, such as the Casein Kinase II (CKII) protein which can also be inhibited by LY294002 [26], we tested the effect of the CKII-inhibitor, DRB (5,6-Dichloro-1-β-D-ribofuranosylbenzimidazole), on trophozoite proliferation. Putative giardial CKII may exist under the accession numbers XP_766966 (for the alpha subunit) and EAA39338 (for the beta subunit), although there is no experimental evidence for this inference. The use of DRB at concentrations known to be inhibitory in mammalian cell types [27] did not cause the same effect on cell proliferation as LY294002 (Figure 8b). This, coupled with the fact that relatively low concentrations of LY294002 cause a significant decrease in cell number, strongly supports a specific effect on putative PI3Ks. Furthermore, this data suggests that trophozoite proliferation is dependent on the functionality of PI3K signalling.

## Discussion

Our study has identified and characterised two putative and distinctive giardial PI3K-encoding genes and gene products. GiPI3K1 is predicted to be a Class I PI3K and GiPI3K2 a Class III PI3K; both are predicted to be functional as PI3Ks and both are expressed during normal growth and possibly during encystation. In addition, we have demonstrated that inhibition of putative giardial PI3Ks by the PI3K inhibitor LY294002 causes a specific and significant inhibition of trophozoite proliferation.

Interestingly, the PI3K inhibitor wortmannin did not effect trophozoite growth as LY294002 did. This was despite our attempts to account for wortmannin instability [28,29] by both testing the activity of our stock solutions on mammalian cell cultures to detect reduced phosphorylated PKB levels and by making repeated additions of the agent to trophozoite cultures (data not shown). Wortmannin-insensitive PI3Ks have been described in yeast, where yeast Vps34 is known to be 1200× less susceptible to wortmannin inhibition than its human homologue [30]. Differences in sensitivity can be explained by differences in key ATP/wortmannin-binding regions of the respective Class III isoforms [30]. Analyses of the giardial PI3Ks demonstrate that they too have residues that differ at the same positions described for yeast Vps34. For example, human Vps34 and yeast Vps34 differ at positions equivalent to positions Ile-831 and Gly-868 in the *S. scrofa *sequence in Figure 3. At these positions, the isoleucine is replaced by a leucine and the glycine replaced with a serine in both GiPI3K1 and GiPI3K2. These residue differences might explain *Giardia*'s apparent insensitivity to wortmannin. An alternative explanation is that the insertions located near to the active sites of GiPI3K1 and GiPI3K2 somehow interfere with wortmannin binding, but not LY294002 binding, since the inhibitory mechanisms of both inhibitors are quite distinct. Wortmannin is an irreversible inhibitor that causes conformational changes to the PI3K active site, whereas LY294002 is a reversible inhibitor that binds less deeply into the active site than wortmannin [30]. Further analysis will be required to test the wortmannin sensitivity of purified giardial PI3Ks.

The giardial PI3Ks contain long insertions which effectively appear to separate the two lobes of their putative kinase domains. These insertions are unique to the giardial sequences and are likely to form part of the protein sequence, as they are not intron-derived. The reason for their existence is unknown, but could simply represent a peculiar feature of the evolutionarily distant/parasitically-divergent *G. intestinalis*. The presence of the giardial PI3K insertions further adds to the observation of relatively large insertions of unknown function found in protein kinases from other organisms such as *Plasmodium*, *Leishmania *and trypanosomes [31] in addition to other giardial sequences [32]. Insertions in giardial sequences have been suggested to serve as useful drug targets [32]. This is particularly relevant in the case of the giardial PI3Ks identified here since PI3K activity in *G. intestinalis *is important for parasite proliferation. Hence drugs that could selectively target giardial PI3Ks on the basis of their insertion sequences would be highly desirable in terms of being parasite-specific and effective. Current progress in designing PI3K-directed pharmacological agents [33] suggests that this could be attainable in the future.

**Figure 9:**
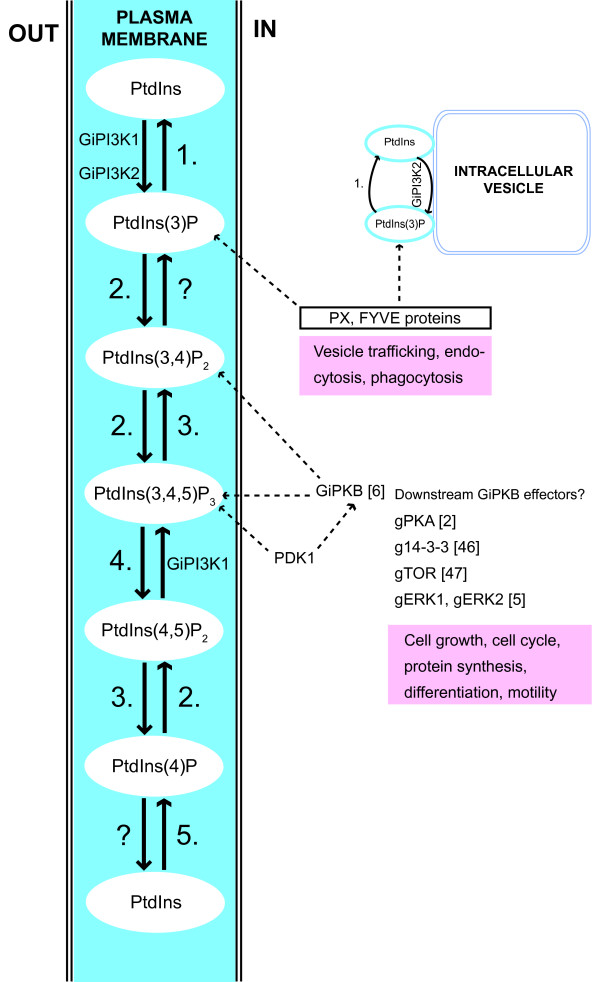
**Proposed model for PI metabolism and PI3K signalling in *G. intestinalis***. A model of PI3K signalling in *G. intestinalis *based on known mechanisms in mammalian cells and yeast. GiPI3K1 and GiPI3K2 are suggested to localise at the plasma membrane and GiPI3K2 at some undefined intracellular compartment. Proteins that may participate in *G. intestinalis *PI metabolism have been identified and are shown (as indicated by numbering): (1) MTM (Myotubularin, GenPept: EAA37232 or EAA38552), (2) PIPkin (Phosphatidylinositol phosphate kinase, GenPept: EAA42556), (3) PI 5-phosphatase (GenPept: EAA40147), (4) PTEN (Phosphatase and tensin homolog, GenPept: EAA42276), (5) PI4K (Phosphoinositide 4-kinase, GenPept: EAA38121). The question mark indicates a possible 4-phosphatase of unknown identity. PtdIns(3)P possibly recruits PX and FYVE -domain-containing proteins, of which there are several encoded in the *G. intestinalis *genome (PX-domain proteins: EAA39579, EAA42255, EAA42811, EAA42799 are possible sorting nexins; FYVE-domain protein: EAA42608, a possible endofin). A putative giardial PDK1 (3-phosphoinositide-dependent protein kinase, GenPept: EAA38977) may be recruited to PtdIns(3,4,5)P_3_. Other previously characterised giardial signalling components which may be invoked could include: GiPKB [6], gPKA [2], g14-3-3 [46], gTOR [47] and gERK1 and gERK2 [5]. Not shown in this figure is the possible generation of PtdIns(3,4)P_2 _from PtdIns(4)P by GiPI3K1.

The identity of Class I and Class III isoforms suggests that *G. intestinalis *is capable of PI-based signal transduction for a range of processes covering typical Class I PI3K sites (the plasma membrane and cytosol for receiving and processing extracellular signals) and Class III PI3K sites (intracellular membranes for regulation of vesicle trafficking). To understand the potential role of PI3K signalling in *G. intestinalis *in a broader context, we used sequence-similarity tools to identify additional giardial signalling homologues. Figure 9 shows giardial proteins which could metabolise the putative products of GiPI3K1 and GiPI3K2 in addition to other PI intermediates, starting with phosphatidylinositol which has already been found in *G. intestinalis *[8]. The PI-metabolising phosphatases identified include: a PI 5-phosphatase and a PTEN-like PI 3-phosphatase which may both act to dephosphorylate the likely products of GiPI3K1 in addition to an MTM-like PI 3-phosphatase that could dephosphorylate the likely product of GiPI3K1 and GiPI3K2. The PI-metabolising kinases identified include: a PIPkin, or phosphatidylinositol-phosphate kinase of as yet unknown type which could phosphorylate several PI-intermediates, such as PtdIns(3)P, PtdIns(3,4)P_2 _and PtdIns(4)P in addition to a putative PI-4 kinase to phosphorylate PtdIns for the purpose of eventual PtdIns(3,4,5)P_3 _generation. Thus sequence analysis does support mechanisms for metabolising the products of the giardial PI3Ks.

To predict how these products, PtdIns(3)P, PtdIns(3,4)P_2 _and PtdIns(3,4,5)P_3_, may act as effective second messengers for downstream effectors, we searched the *G. intestinalis *genome [16] for proteins which have the relevant PI-binding domains such as phox-homology (PX), FYVE (*F*ab1, *Y*OTB/ZK632.12, *V*ac1, and *E*EA1) and pleckstrin-homology (PH). PH-domain containing proteins such as protein kinase B (PKB) use their PH domain to facilitate recruitment to the plasma membrane where PtdIns(3,4,5)P_3 _is located. Although Kim et al. (2005) failed to identify a PH domain in the giardial PKB, GiPKB, with significant similarity to the PH domain of other PKBs, the percentage identity and similarity scores following our own sequence alignment of GiPKB's putative PH domain with that of human PKB were 12 and 36 % respectively (data not shown). Thus it may be that GiPKB is capable of binding PtdIns(3,4,5)P_3 _via a putative divergent PH domain. Another protein that binds PtdIns(3,4,5)P_3 _via its PH domain is 3-phosphoinositide-dependent kinase-1 (PDK1) which phosphorylates PKB. We were able to identify a putative giardial PDK1 homologue which did not have an easily identifiable PH domain when employing domain-prediction tools. However upon pairwise-alignment of its sequence with that of human PDK1, a PH domain-like sequence was identified with 23 % identity and 51 % similarity (data not shown).

Figure 9 proposes that from GiPKB, several other regulatory pathways involving protein components previously characterised in *G. intestinalis *could be initiated. We were able to identify PX- and FYVE-domain containing proteins encoded by the *G. intestinalis *genome [16]. In mammalian cells these domains facilitate binding and recruitment to PtdIns(3)P. The giardial PX-domain proteins tend to be similar to sorting nexins, and the FYVE-domain protein is similar to endofin. Both of these proteins are implicated in regulating membrane traffic. Thus via these giardial homologs it may be possible to connect the product of GiPI3K2 with vesicle trafficking mechanisms. Overall, the presence of putatively functional PI3Ks, PI-metabolising enzymes and downstream effectors further substantiates the existence of *bona-fide *PI3K signalling pathways in *G. intestinalis*, as supported by our functional data employing a widely-used and characterised PI3K inhibitor.

## Conclusion

We conclude that the human intestinal parasite *G. intestinalis *encodes and expresses two putative PI3Ks, GiPI3K1 and GiPI3K2, both of which are likely to be functional based on sequence analysis. Functionality of PI3K-dependent processes has been demonstrated by observing the specific inhibitory effect of a PI3K-inhibitor on trophozoite proliferation. Phylogenetic analysis supports the assignment of GiPI3K1 and GiPI3K2 to the Class I and Class III PI3K subdomains respectively, and in-depth sequence analysis further substantiates their proposed substrate specificities. Genome sequence analysis can link the products of GiPI3K1 and GiPI3K2 with putative giardial PI-metabolising phosphatases and kinases, in addition to downstream effectors with PI-binding domains. This provides additional evidence for *bona-fide *PI3K signalling pathways in *G. intestinalis*.

A feature unique to both giardial PI3Ks is the presence of a large insertion in a highly conserved region of their kinase domains. Both insertions are not subject to splicing and are not predicted to interfere with substrate binding. Thus, considering the important role of PI3Ks for parasite proliferation, it is possible that the insertion sequences could be exploited for drug-targeting purposes.

## Methods

### Sequence similarity searches and alignments

The translation BLAST [34] tool at the National Center for Biotechnology Information (NCBI) was used with default settings to search for putative PI3Ks in the *Giardia intestinalis *genome [16] using the kinase domain sequences of the following protein sequences as queries: *Homo sapiens *p110α [GenPept: NP_006209], *H. sapiens *PI3K-C2α [GenPept: NP_002636] and *Saccharomyces cerevisiae *Vps34 [GenPept: NP_013341]. The searches returned several matches to putative open reading frames (ORFs). Gene sequences in a non-redundant database at NCBI were searched for similar sequences to the putative ORFs via NCBI's translation-BLAST and nucleotide-BLAST tools (used with default settings) in order to identify two putative PI3K-encoding ORFs (subsequently called *Gipi3k1 *and *Gipi3k2*). SMART [35], and SCOP [36] were used with default settings to predict PI3K-characteristic domains in GiPI3K1 and GiPI3K2 and other PI3Ks. Several sequences were multiple-aligned using ClustalW [37] with default settings. Secondary structure predictions were carried out using the PSIPRED server [38] used with default settings.

### Phylogenetic analysis

The conceptually translated *G. intestinalis *PI3K sequences, GiPI3K1 and GiPI3K2, were aligned to reference sequences from Genbank using ClustalW [37]. In order to obtain a taxonomically sound sample, homologues sequences were sought amongst the Metazoa, Viridiplantae, Fungi, Stramenopiles, Alveolates, and species generally considered to be 'deep-branching'. The alignments were manually refined and only unambiguously aligned regions without gaps were used for phylogenetic analysis, leaving a dataset of 26 taxa with 473 amino acid positions. Likelihood searches were performed in a Bayesian framework using mixed amino acid models accommodating site rate variation (fraction of invariable sites plus eight variable gamma rates) using the program MrBayes [39]. All analyses started with randomly generated trees and ran for 100,000 generations, with sampling at intervals of 100 generations that produced 1,000 trees. The log-likelihood values of the 1,000 trees were plotted against the generation time (not shown). All the trees produced prior to reaching stationarity were discarded, making sure that burn-in samples were not retained. Although the likelihood model stabilized very rapidly (data not shown), only the last 900 trees were used to estimate separate 50% majority rule consensus trees for these. The frequency of any particular clade, among the individual trees contributing to the consensus tree, represents the posterior probability of that clade [39]. In addition, maximum likelihood (ML) analyses were obtained using PHYML [40]. The protein data set was re-sampled 100 times and analysed with alpha and invariant sites parameters optimised on the initial BIONJ tree produced by PHYML. Analyses were done with a mixed eight-category discrete gamma plus invariable sites model of rate heterogeneity. The JTT substitution model was used in the protein analyses. Majority rule consensus trees were obtained from the resulting 100 trees.

### G. intestinalis cell culture

*G. intestinalis *strain WB (ATCC 30957, clone C6) was routinely subcultured every 3 to 4 days at 37°C in axenic modified YI-S medium [41]. Encystation was carried out according to published procedures [42].

### PI3K inhibition

Routinely subcultured *G. intestinalis *trophozoites were grown for 48 hours prior to treatment with the PI3K inhibitor LY294002 (2-(4-Morpholinyl)-8-phenyl-4H-1-benzopyran-4-one hydrochloride) and CKII inhibitor DRB (5,6-Dichloro-1-β-D-ribofuranosylbenzimidazole). Cells were treated with LY294002 to achieve final concentrations of 25, 50, 75, 100 and 200 μM from a 100× stock solution in DMSO. Cells were treated with DRB to achieve a final concentration of 100 μM from a 100× stock solution. Control cells were treated with equal volumes of DMSO. Experiments were carried out in triplicate. Cells were fixed in 4% paraformaldehyde (Sigma) and counted with a haemocytometer. Count data was tested for normal distribution and significance using the Student t-test within the SigmaPlot package (Systat Software Inc.).

### Nucleic acid methods

#### DNA and RNA isolation

Genomic DNA was extracted from harvested trophozoites according to the TriPure Isolation method (Roche). Total RNA was extracted from both harvested trophozoites and encysting cells using the RNAeasy Mini Kit for animal cells (Qiagen). RNA was purified from contaminating DNA using *DNase *I (Invitrogen) and the RNAeasy Mini Protocol for RNA Cleanup (Qiagen) was used to remove components of the *DNase *I digestion reaction from RNA.

#### Generation of cDNA

Total RNA was reverse-transcribed into cDNA in a 20 μl reaction mix containing 6 μg total RNA, 5 μM random hexamer primers, 2.5 mM DTT, 250 μM dNTPs, 40 U RNaseOUT recombinant ribonuclease inhibitor (all from GibcoBRL) and 25 U MLV RT (GeneSys Ltd). RNA was denatured by heating at 70°C 10 min, followed by cooling on ice to anneal random hexamer primers. MLV RT was added, and incubated at 37°C for 1 hour. Negative controls replaced RT with RNAse-free water.

#### Polymerase Chain Reaction (PCR)

PCR was performed on *G. intestinalis *cDNA, genomic DNA and plasmid DNA using the following gene specific primers: *Gipi3k1 *ins_5465S: (5' GCC ATT CGA GGG TTT GAT AG 3'); *Gipi3k1 *ins_6216AS: (5' GAT TGC TAG TAG GGC ATC TG 3'); *Gipi3k2 *4871AS: (5' TGT GGA AGG CGT CGA TGA AC 3'); *Gipi3k2 *ins_3424S: (5' ACG ATG TGC GAC TAG ACA TG 3'); *Gipi3k1*(1)*Not*I S: (5' GAG AGC GGC CGC ACC ATG GGA ATG CAG GTT TCC ATT ATA AG 3'); *Gipi3k1*(1)*Bam*HI AS: (5' CTC TGT AGG TGG CTT ATA TG 3'); *Gipi3k1*(2)*Bam*HI S: (5'CTA TGC GCA TAA GCT TTG AG 3'); *Gipi3k1*(2) *Bam*HI AS (5' GGA AGC GCC TCC TCC AAC TCT AAG GAT CCG AGA 3'). Primer sequences designed to demonstrate the presence of the intron in some *G. intestinalis *ferredoxin-encoding mRNAs were taken from Nixon et al. (2002) [43] and included as a positive control to confirm the presence of spliced and unspliced mRNA. *Gipi3k1 and Gipi3k2 *PCR products (generated from primers *Gipi3k1 *5945S/6374AS and *Gipi3k2 *4441S/4871AS respectively) were cloned into pGEM-T Easy (Promega) and sequenced using the dideoxy-nucleotide termination method at the DNA Analysis Facility, Dundee University. Sequence output was analysed and verified using BioEdit [44].

#### Rapid Amplification of cDNA Ends (RACE)

The 5' RACE system from Invitrogen was used to determine the start of *Gipi3k1 *transcription. Oligonucleotides GSP1_393 (5' GCT GAC AAG AGC CTC TGC TA 3') or GSP1_2947 (5' GGC TTT AGA AGA AAC AAG GA 3') primed the first strand cDNA synthesis, and oligonucleotides GSP2_313 (5' GCC AGC TTT CTA GCA GGT CA 3') or GSP2_2914 (5' GTC CAC TTG AGA AGT ATC CT 3') served as nested primers. PCR products were cloned and sequenced as above.

#### Northern blotting

Northern blotting was carried out using a nonradioactive method (Roche). DNA probes incorporating digoxigenin (DIG) were generated against the kinase-domain encoding region of *Gipi3k1*/*2 *via PCR amplification of pGEM-T Easy plasmids (Promega) carrying sequences of *Gipi3k1*/*2 *genes. 5 μg *G. intestinalis *total RNA was prepared as described above and separated on a 1.2 % agarose/0.5 % formaldehyde/1% 1× MOPS (3-M-Morpholino propanesulphonic acid) gel and transferred to positively charged nylon membrane (BrightStar-Plus, Ambion) by capillary transfer. RNA was fixed to the membrane by baking at 80°C for 2 hr. DIG-labelled DNA probes were hybridised to blots for 16 hours at 50°C. Membranes were washed at high stringency using techniques described by Roche. Antibody solution (1:10,000 Anti-Digoxigenin-AP) (Roche, 75 mU/ml) was added to the membrane for 30 min at room temperature. Chemiluminescent substrate CSPD (Roche) was added to the membrane and exposed to film.

### Cloning and sequencing of Gipi3k1

The entire *Gipi3k1 *nucleotide sequence was cloned into the pcDNA 3.1/myc-His(-) B vector (Invitrogen) as follows: the 6,468 bp *Gipi3k1 *gene sequence was PCR-amplified using *Pfu *polymerase from *G. intestinalis *genomic DNA as two approximately-equal sized inserts using primer pairs as described above which would incorporate *Not*I and *Bam*HI restriction sites, specifically 5' *Not*I - 3' *Bam*HI for the first insert and 5' *Bam*HI - 3'*Bam*HI for the second. Inserts were ligated into *Not*I/*Bam*HI (Promega) linearised vector using T4 DNA ligase (Promega), transformed and cloned in SURE *Escherichia coli *(Stratagene) cells and plasmid DNA extracted using a Qiagen miniprep kit. pcDNA3.1/myc-His(-) B - *Gipi3k1 *plasmid was sequenced across the entire length of the *Gipi3k1 *sequence as described above.

## Abbreviations

PI3K: Phosphoinositide-3 kinase; PI4K: Phosphoinositide-4 kinase; PtdIns: Phosphatidylinositol; PI: Phosphoinositides; PtdIns(3)P: PtdIns-3-phosphate; PtdIns(4)P: PtdIns-4-phosphate; PtdIns(3,4)P_2_: PtdIns-3,4-bisphosphate; PtdIns(4,5)P_2_: PtdIns-4,5-bisphosphate; PtdIns(3,4,5)P_3_: PtdIns-3,4,5-trisphosphate; PIPkin: Phosphatidylinositol phosphate kinase; MTM: Myotubularin; PTEN: Phosphatase and tensin homolog; RT-PCR: Reverse Transcriptase Polymerase Chain Reaction; 5' RACE: 5' Rapid Amplification of cDNA Ends; RBD: Ras Binding Domain; GiPI3K1/2: *Giardia intestinalis *PI3K1/2; CKII: Casein Kinase II; DRB: 5,6-Dichloro-1-β-D-ribofuranosylbenzimidazole; LY294002: 2-(4-Morpholinyl)-8-phenyl-4H-1-benzopyran-4-one hydrochloride; PX: Phox homology; PDK1: 3-phosphoinositide-dependent kinase-1; PKB: Protein kinase B; PH: Pleckstrin homology; FYVE: *F*ab1, *Y*OTB/ZK632.12, *V*ac1, and *E*EA1.

## Authors' contributions

SSEC carried out the sequence alignments, annotation and domain characterization, in addition to the cell culturing, molecular genetic and inhibition studies. SSEC also drafted the manuscript. MvdG carried out the phylogenetic analysis. SJT contributed toward the inhibition studies. All authors participated in the sequence alignments and domain characterization. MRC and JT conceived of the study, and participated in its design and coordination and helped to draft the manuscript. All authors read and approved the final manuscript.

## Figures and Tables

**Table 1:** Analysis of the giardial PI3K domains. Percentage identity values for the full-length and domain-only sequences of GiPI3K1 and GiPI3K2 with other PI3K isoforms following their sequence alignment.

	**GiPI3K1**	**GiPI3K2**
**Full- length**	39% to *Dictyostelium discoideum *PIK1, a Class I PI3K	34% to *Glycine max *Vps34-like (Class III) PI3K
**Ras Binding Domain (RBD)**	41% to *Homo sapiens *Class IB p110γ PI3K	Absent
**PIK**	28% to *H. sapiens *Class IA p110α PI3K	27% to *Caenorhabditis elegans *Vps34-like (Class III) PI3K
**Kinase**	39% to *D. discoideum *PIK1	33% to *Glycine max *Vps34-like (Class III) PI3K
